# Decreased Severity of Acute Hepatopancreatic Necrosis Disease in White Shrimp (*Litopenaeus vannamei*) by Mixed Culture of *Bacillus subtilis*, *Bacillus licheniformis* and *Bacillus megaterium*

**DOI:** 10.21315/tlsr2023.34.1.6

**Published:** 2023-03-31

**Authors:** Saowapha Surawut, Kunyarut Suntara, Winyou Puckdee, Chutapa Kunsook, Pornpimon Kanjanavas, Anchalee Kompatiparn, Prachuab Leeruksakiat

**Affiliations:** 1 Department of Biology, Faculty of Science and Technology, Rambhai Barni Rajabhat University, Chanthaburi 22000, Thailand; 2 Kung Krabaen Bay Royal Development Study Center, Chanthaburi 22120, Thailand; 3 Faculty of Nursing, Kasem Bundit University, Bangkok 10510, Thailand

**Keywords:** Acute Hepatopancreatic Necrosis Disease, *Litopenaeus vannamei*, *Bacillus subtilis*, *Bacillus megaterium*, *Bacillus licheniformis*

## Abstract

The objective of this study was to investigate the mixed culture of *Bacillus subtilis, B. licheniformis and B. megaterium* to control acute hepatopancreatic necrosis disease (AHPND) or EMS (Early Mortality Syndrome) in white shrimp *Litopenaeus vannamei* as a model. The infected shrimps with *Vibrio parahaemolyticus* AHPND strain were divided into tanks and different feeding of either *B. subtilis, B. licheniformis*, *B. megaterium* or all *Bacillus* strains. The infected shrimps that were fed with a mixed culture of *Bacillus* showed significantly highest survival rate and revealed lower percent detection of *V. parahaemolyticus* AHPND strain by Polymerase Chain Reaction (PCR) (57.14%) with a small amount of viability count in their hepatopancreas. In contrast, the infected shrimps that were fed with each of *B. subtilis, B. licheniformis* or *B. megaterium*, revealed the spread of *V. parahaemolyticus* AHPND strain in all tissue by PCR detection (86.67%–100%) with a large amount of viability count (3.53 – 4.24 × 10^3^ CFU/g). This study indicated that the mixed culture of *Bacillus subtilis, B. licheniformis and B. megaterium* could control the dissemination of *V. parahaemolyticus* in shrimps, especially in hepatopancreatic that is the target tissue of AHPND in white shrimp *(L. vannamei*). The result of this study revealed the efficiency and mechanism of the mixed culture of *B. subtilis, B. licheniformis and B. megaterium* to control the virulence of AHPND and support the application of this mixed culture in aquaculture of shrimp farms to avoid chemical and antibiotic treatment by using it as a biological control.

**Highlights**
- A diet supplemented with the mixed culture of *Bacillus subtilis*, *B. licheniformis*, and *B. megaterium* could decrease AHPND severity in white shrimp (*L. vannamei*).
- Infected shrimp fed with a diet supplemented with the mixed culture of *Bacillus* strain revealed higher survival percentage, lower percent of *Vibrio* AHPND strain detected by PCR, and a small amount of *Vibrio* sp. viability count in hepatopancreas than those other groups of infected shrimps.
- The mixed culture of these *Bacillus* strains can control the dissemination of the *Vibrio* AHPND strain to the hepatopancreas as a target tissue of AHPND.

## INTRODUCTION

White shrimp (*Litopenaeus vannamei*) is one of the significant economic seafood products. However, the disease outbreak in the shrimp farming industry caused by microbial pathogens results in losses of production. Acute Hepatopancreatic Necrosis Disease (AHPND) also known as Early Mortality Syndrome (EMS) commonly affects shrimp post-larvae or juveniles and leads to 100% shrimp death within 30 days to 35 days after stocking (Velázquez-Lizárraga *et al*. 2019; De Schryver *et al*. 2014). The causative agent of AHPND is the *Vibrio parahaemolyticus* AHPND strain, the gram-negative bacteria with the halophilic property and toxin production. This pathogen has a plasmid harbouring virulent genes that encode homologous of the Photorhabdus insect-related (Pir) toxins, PirA and PirB (or PirAB^VP^) (Tinwongger *et al*. 2014). The route of infection is the exposure of mouth, gills, feed and tissue damage to *V. parahaemolyticus* AHPND strain (Prachumwat *et al*. 2019; Lai *et al*. 2015). Subsequently, the disease develops by defects in the immune system and a large amount of this pathogen in their environment. The severity of AHPND has led to the study of how to prevent and treat this disease. The use of microbial culture as a probiotic in the treatment of organic waste in water and soil to reduce the risk factor of *V. parahaemolyticus* AHPND strain proliferation has been increasingly interesting (Kumar *et al*. 2016; Hlordzi *et al*. 2020). The genus of *Bacillus* has previously been reported as a potential probiotic supplemented in a diet for shrimp. A diet containing *B. subtilis* was fed to *L. vannamei* and demonstrated better growth than a non-supplemented control group (Liu *et al*. 2009). Additionally, *B. subtilis, B. licheniformis* and *B. megaterium* could inhibit the growth and toxin production of *Vibrio harveyi* leading to a higher survival rate of shrimp with a diet containing these probiotics than those in a control group with non-supplemented (Nakayama *et al*. 2009). Moreover, aquaculture of shrimp farms with probiotics demonstrated a better quality of water in the pond (Kumar *et al*. 2016; Hlordzi *et al*. 2020). However, little is known about the mechanism of these *Bacillus* species and *V. parahaemolyticus* AHPND strain in host interaction. The purpose of this study was to investigate the effect of *B. subtilis, B. licheniformis, B. megaterium* and their mixed culture on the disease severity of AHPND in the infected shrimp model.

## MATERIALS AND METHODS

### Experimental Shrimp

Healthy shrimp (*L. vannamei*, PL21) were provided by Kung Krabaen Bay Royal Development Study Center, Chantaburi, Thailand. They were maintained in the tank (500L) at 25°C–27°C, the salinity of 23 ppt. with aeration, and were fed commercial feeds two times a day. Some shrimps were collected to confirm uninfected of *V. parahaemolyticus* by PCR detection (Tinwongger *et al*. 2014).

### Pathogen Inoculum Preparation

The *V. parahaemolyticus* AHPND strain was kindly provided by Kung Krabaen Bay Royal Development Study Center, Chantaburi, Thailand. This bacterial strain was originally isolated from the shrimp farm in Chantaburi. Confirmation of the virulence *V. parahaemolyticus* AHPND strain was performed using the challenge test and histopathological examination as previously described (Tinwongger *et al*. 2014). The bacteria were single colonies isolated on thiosulfate citrate bile-salt sucrose (TCBS) agar and grow in tryptic soy broth (TSB) supplemented with 2% NaCl at 37°C for 18 h on an incubator shaker. The media was removed by centrifugation at 10,000 rpm for 1 min. The bacterial cells were resuspended in normal saline for absorbance measurement. An absorbance value of 1 at 540 nm, corresponds to a cell density of approximately 10^9^ CFU/mL. The bacterial concentration at 10^8^ CFU/mL was used as the infective dose as previously described (Khimmakthong & Sukkarun 2017).

### *Bacillus* Inoculum and Feed Preparation

*Bacillus subtilis*, *B. megaterium* and *B. licheniformis* were purchased from the Thailand Institute of Scientific and Technological Research (TISTR). These bacterial strains were cultured on nutrient agar (NA) and incubated at 35°C for 24 h. Subsequently, starter cultures were prepared by inoculating one loopful of each strain in 40 mL of nutrient broth (NB) with a magnetic stirrer and incubated at room temperature for a day. The mixed culture of *Bacillus* strains was performed by adding 40 mL of each strain in 4 L of minimal medium (MM) and cultured for 36 h to 72 h at room temperature. A pure culture of each strain was prepared by using 120 mL of each strain in 4 L of MM. The total plate counts were performed to determine the number of viable bacterial cells in the microbial culture. Either pure culture or mixed culture of these *Bacillus* strains was prepared in 10^7^ CFU/mL to supplement feeding. Feed preparation by adding 5 mL of each bacterial suspension (10^7^ CFU/mL) to 10 g of commercial feeds, mixed, and air-dried. The controlled feeding was only commercial feeds that did not mix with any *Bacillus* strain.

### *Vibrio parahaemolyticus* Infection Model with *Bacillus* sp. Treatments

To investigate the effect of *Bacillus* culture on controlling the severity of AHPND in infected shrimps. All shrimp were infected with *V. parahaemolyticus* AHPND strain as previously described (Khimmakthong & Sukkarun 2017). Briefly, approximately 300 shrimps were immersed in seawater containing *V. parahaemolyticus* AHPND strain at 10^8^ CFU/mL for 1 h. After exposure, the shrimp were washed with sterile seawater and placed in new sterile seawater without seawater exchange. In the uninfected group, 60 shrimps were no *V. parahaemolyticus* AHPND strain treatment. The infected shrimps were divided into 60 shrimps per tank for 5 tanks and each tank contained 75 L of seawater. The infected shrimps were cultured with normal feed (control+*V.parahaemolyticus*) (tank 1), feed supplement with a mixed culture of *B. subtilis*, *B. megaterium* and *B. licheniformis* (BS+BL+BM) (tank 2), feed supplement with *B. subtilis* (BS) (tank 3), feed supplement with *B. licheniformis* (BL) (tank 4), and feed supplement with *B. megaterium* (BM) (tank 5). Additionally, the 60 uninfected shrimps were treated with normal feed in tank 6 (control-*V. parahaemolyticus*). All tanks were fed 10 g of feeding diet per tank, three times a day. After infection, tanks were visually monitored every day. Dead shrimp were collected for DNA extraction to confirm AHPND strain infection by PCR and recorded indicating the time at which mortality occurred. For survival analysis, the shrimps were observed for 22 days after *V. parahaemolyticus* administration, and percentage of survival was calculated by dividing the number of survival shrimp by the initial number of shrimps × 100. At the endpoint of the experiment, all survival shrimps were sacrificed 22 days after *V. parahaemolyticus* administration. At the time of euthanasia by lacking oxygen, the various tissues (hepatopancreas, muscle, and intestine) were collected for DNA extraction and were fixed with 70% ethanol.

### Detection of *V. parahaemolyticus* AHPND strain by PCR

DNA extraction was performed using the boiling method. Briefly, the shrimp tissue was homogenised by micropestle in a 1.5 mL microcentrifuge tube with vortex regularly and 900 μL of TSB supplement with 2% NaCl were added and incubated at 37°C for 24 h to 48 h. Subsequently, aliquoted 700 μL of culture media was in a new tube and then centrifuged at 12,000 rpm for 5 min. The cell pellet was collected for the extraction of DNA. The 200 μL of distilled water was added to the cell pellet for resuspending. The cell suspension was boiled for 5 min and centrifuged at 12,000 rpm for 5 min. The supernatant was used as a DNA template and stored at −20°C until used. The DNA extraction of bacterial pure culture was performed by resuspending a single colony in 200 μL of distilled water in a microcentrifuge tube. The bacterial suspension was boiled for DNA extraction as described above. The set of primers used in this study was shown in Table 1. Two primers sets were used to detect the plasmid that was specific for AHPND strain (TUMSAT-Vp1F, TUMSAT-Vp1R, and TUMSAT-Vp3F, TUMSAT-Vp3R), and species-specific primer set, flaE gene or flagella gene in *V. parahaemolyticus* chromosome (Vp-flaE-79F, Vp-flaE-34R) were used as previously described (Tinwongger *et al*. 2014). A tube of the PCR reaction (20 μL) included 2 μL of DNA template, 2.4 μL of 10 μM Forward/Reverse Mix primer (Vp-flaE, Vp1, Vp3), 10 μL of 2x PCR Master Mix (i-TaqTM plus, Korea) and 5.6 μL PCR grade-water (Invitrogen, USA). All PCR was conducted under the following condition: initial denature at 94°C for 3 min, then amplified for 29 cycles at 94°C for 30 s, 60°C for 30 s, and 72°C for 30 s. The cycling was terminated at 72°C for 2 min (Tinwongger *et al*. 2014). PCR products were determined by gel electrophoresis on 1.5% agarose gel with RedSafe (iNtRON, Korea) at 100 V for 30 min. The percent of *V. parahaemolyticus* detection in tissue was calculated as the number of detected tissues divided by the number of all tissue × 100.

### The Viability Count of *Vibrio parahaemolyticus* in Tissue

The amount of viable *V. parahaemolyticus* in the tissue of shrimp was performed on TCBS agar. Briefly, the tissues were weighed and grounded by using a micropestle. Ten-fold serial dilution was performed and spread on TCBS agar plates. All plates were incubated at 35°C for 24 h to 48 h. Only green colonies were counted in colony-forming units per gram of tissue (CFU/g).

### Determination of Water Quality Parameter

The water in all experimental tanks was sampled for determination of water quality by pH, alkalinity (mg/L), salinity (ppt), ammonia (mg/L), nitrite (mg/L) and dissolved oxygen (DO) (mg/L) as previously described (Kyeong-Jun *et al*. 2019).

### Statistical Analysis

The mean ± SD was used for data presentation. Survival analyses were evaluated with the log-rank test. The statistically significant difference between two groups and more than two groups was examined by the *t*-test and one-way analysis of variance (ANOVA) with Tukey’s multiple comparisons, respectively. The *p-*values < 0.05 were considered statistically significant. SPSS 11.5 software (SPSS Inc., Chicago, IL, USA) was used for all statistical analyses.

## RESULTS

### Decreased Severity of Hepatopancreatic Necrosis Disease in White Shrimp *(Litopenaeus vannamei*) by Mixed Culture of *Bacillus* strain

**Figure 1:**
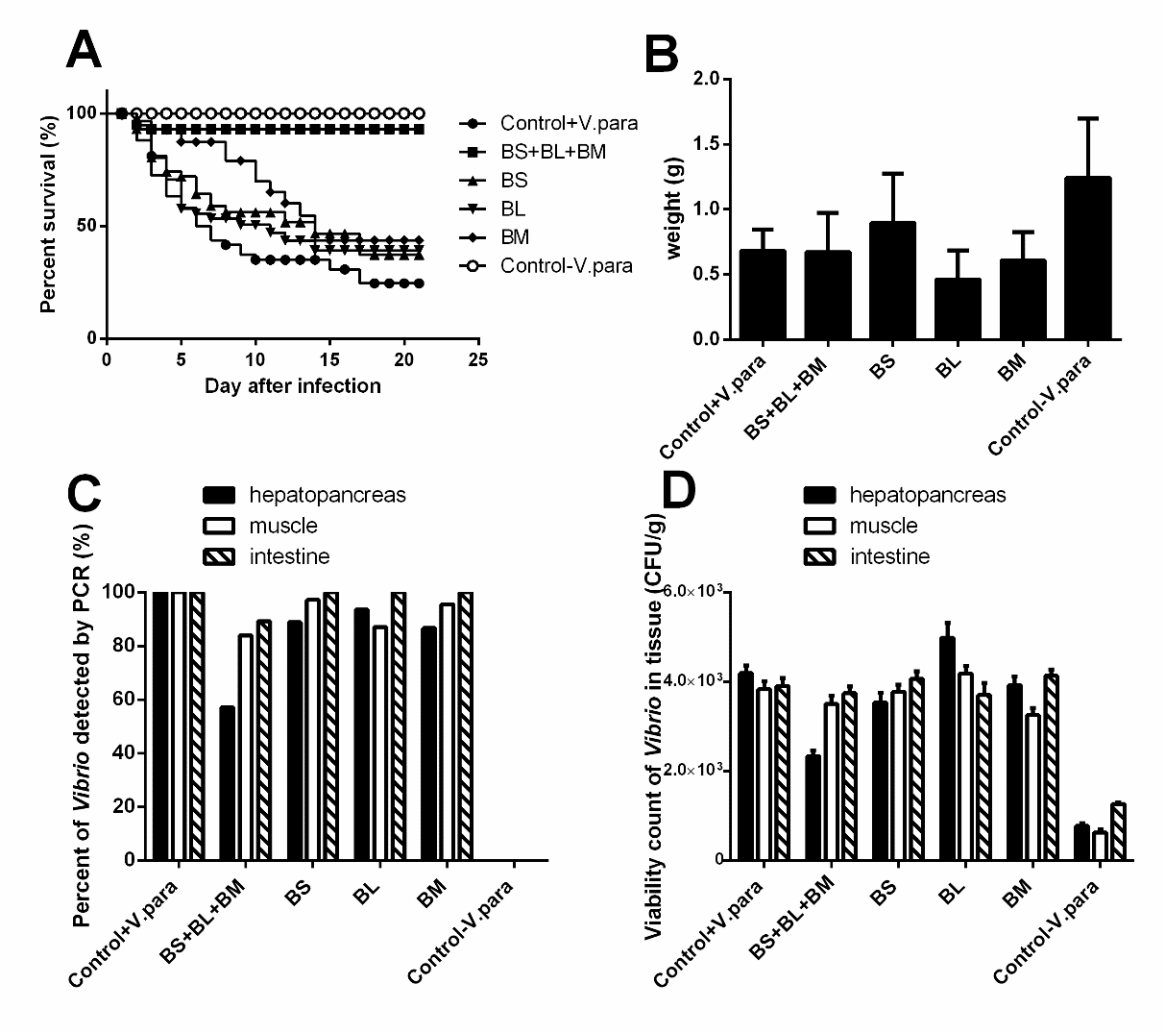
(A) percent survival of *Vibrio*-infected shrimps that feed with various *Bacillus* strains; (B) weight of *Vibrio*-infected shrimps that fed with various *Bacillus* strains after the endpoint of the experiment; (C) percent of *Vibrio*-detected in various tissue by PCR; (D) viability count of *Vibrio* in various tissue.

The infected shrimp were fed with a mixed culture of *B. subtilis, B. licheniformis, and B. megaterium* (BS+BL+BM) revealed a high survival rate but infected shrimp without any bacteria contained in the feeding (Control+*V. parahaemolyticus*) show lower survival rate and significant difference in survival rate between these groups were observed (*p*-value < 0.0001) (Fig. 1A) with indicated that the mixed culture of *Bacillus subtilis* (BS), *Bacillus megaterium* (BM) and *Bacillus licheniformis* (BL) be able to control disease severity of hepatopancreatic necrosis disease in white shrimp by decrease mortality of infected shrimp. However, the survival rate of infected shrimp fed with each strain of *Bacillus sp*. showed no different survival rate with the Control+*V. parahaemolyticus* group except *B. megaterium* (*p*-value 0.0036) and suggested that only one strain of *Bacillus* could not control disease severity and *B. megaterium* maybe play as a key role to control *V. parahaemolyticus* in this infected shrimp model. Additionally, all survival shrimps were weighed at the endpoint of the experiment, the result found that the infected shrimps with and without the *Bacillus* strain contained in the feeding were not significantly different weights (Fig. 1B). However, the weight of uninfected shrimp was higher than all groups of infected shrimps with significant differences (*p*-value < 0.0001).

### The Mixed Culture of *Bacillus* strain was able to Control Disseminated of *V. parahaemolyticus* to Hepatopancreas as a Target Tissue of AHPND

**Figure 2:**
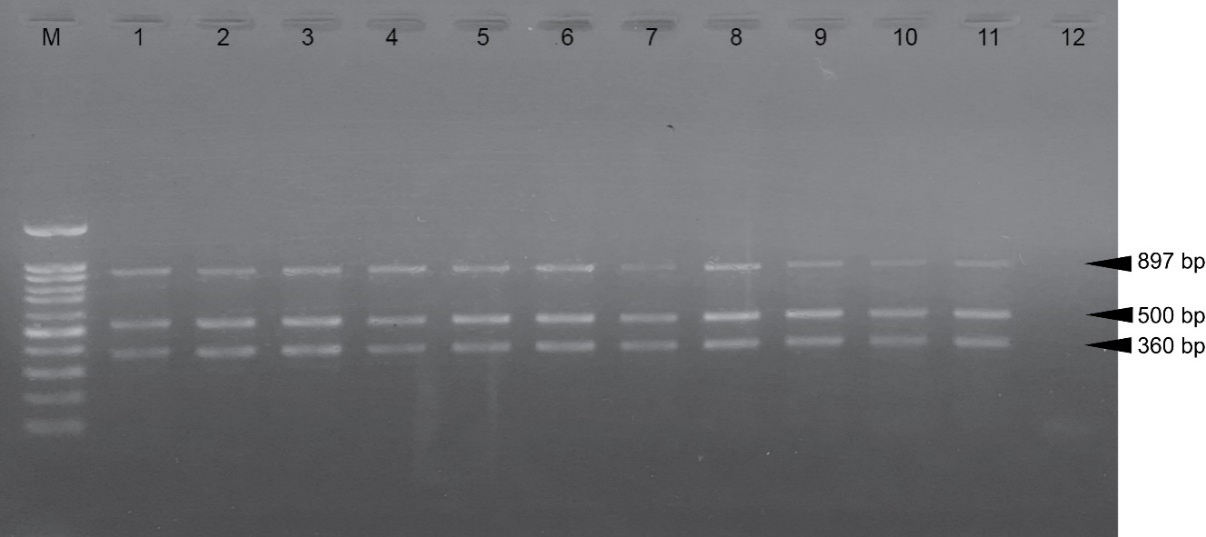
Agarose gel electrophoresis of dead shrimp caused by *V. parahaemolyticus* AHPND strain infection revealed three specific bands consisting of 897, 500 and 360 bp from hepatopancreas specimens. Lane M: 100 pb DNA ladder; Lane 1: positive control (DNA from a pure culture of *V. parahaemolyticus* AHPND strain); Lane 2: sample 1 from tank 1 with normal feed (Control+V. para); Lane 3: sample 2 from tank 1 with normal feed (Control+V. para); Lane 4: sample 1 from tank 2 was feed supplement with the mixed culture of *Bacillus* strain (BS+BL+BM); Lane 5: sample 2 from tank 2 was feed supplement with the mixed culture of *Bacillus* strain (BS+BL+BM); Lane 6: sample 1 from tank 3 was feed supplement with *B. subtilis* (BS); Lane 7: sample 2 from tank 3 was feed supplement with *B. subtilis* (BS); Lane 8: sample 1 from tank 4 was feed supplement with *B. licheniformis* (BL); Lane 9: sample 2 from tank 4 was feed supplement with *B. licheniformis* (BL); Lane 10: sample 1 from tank 5 was feed supplement with *B. megaterium* (BM); Lane 11: sample 2 from tank 5 was feed supplement with *B. megaterium* (BM); Lane 12: negative control (Distilled water).

**Figure 3:**
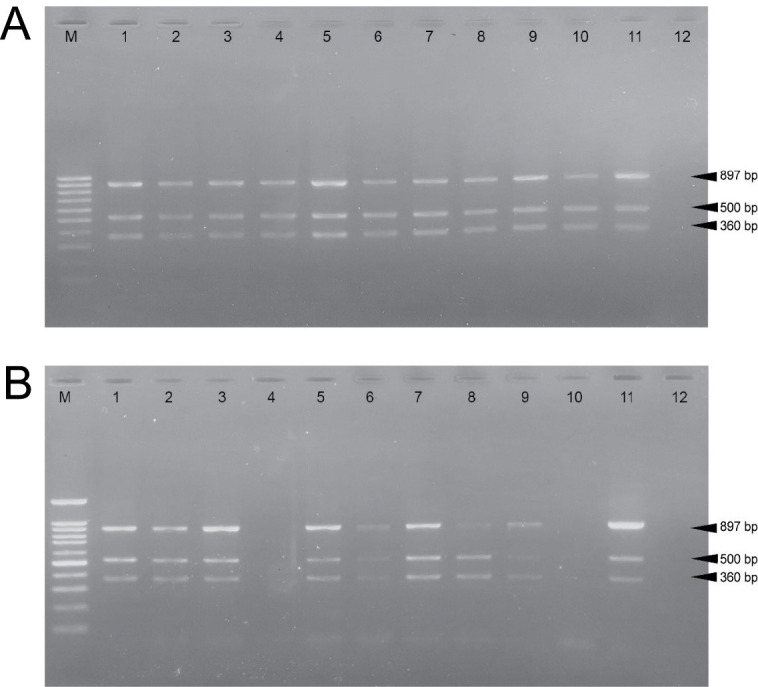
Agarose gel electrophoresis of *V. parahaemolyticus* AHPND strain detection revealed three specific bands consisting of 897, 500 and 360 bp. 3A: The infected shrimp without any bacteria contained in the feeding (Control+*V.parahaemolyticus*). 3B: The infected shrimp were fed with a mixed culture of *Bacillus* sp. (BS+BL+BM). Lane M: 100 pb DNA ladder; Lane 1: positive control (DNA from a pure culture of *V. parahaemolyticus* AHPND strain); Lane 2: hepatopancreas -sample 1; Lane 3: muscle-sample 1; Lane 4: hepatopancreas - sample 2; Lane 5: muscle - sample 2; Lane 6: hepatopancreas - sample 3; Lane 7: muscle - sample 3; Lane 8: hepatopancreas - sample 4; Lane 9: muscle - sample 4; Lane 10: hepatopancreas - sample 5; Lane 11: muscle - sample 5; Lane 12: hepatopancreas – uninfected sample (Control-*V. para*).

To determine the effect of the *Bacillus* strain in controlling the dissemination of *V*. *Parahaemolyticus* in the shrimp model. The hepatopancreas, muscle, and intestine were collected from each infected shrimp and *V*. *parahaemolyticus* AHPND strain was detected by PCR in tissue. All dead shrimp were caused by *V*. *parahaemolyticus* AHPND strain infection that revealed three specific bands consisting of 897, 500, and 360 base pairs (bp) on agarose gel electrophoresis in hepatopancreas (Fig. 2). At the endpoint of the experiment, shrimp were sacrificed to determine *V*. *parahaemolyticus* AHPND strain infection by PCR and the viable count of *Vibrio* sp. in various tissue. The infected shrimp without any bacteria contained in the feeding (Control+*V*. *parahaemolyticus*) reveal all tissues were detected with *V*. *parahaemolyticus* AHPND strain (Figs. 3A, 1C and 1D). The infected shrimp were fed with a mixed culture of *Bacillus* sp. (BS+BL+BM) revealed a lower percent of *Vibrio* detection by PCR (57.14%) and a lower amount of *Vibrio* sp. in hepatopancreas (Figs. 3B, 1C and 1D), while other groups of infected shrimps revealed higher percent of *V*. *parahaemolyticus* AHPND detection and amount of viability of *Vibrio* sp. in all tissue (Figs. 1C and 1D). These results suggested that aquaculture of infected shrimp with the mixed culture of *Bacillus* spp. in feeding (BS+BL+BM) could decrease the severity of AHPND by decreased dissemination of *V*. *parahaemolyticus* AHPND strain to hepatopancreas which is the target tissue of this disease.

### The Water Quality Did Not Affect the Survival of Infected Shrimp During the Experiment

**Figure 4:**
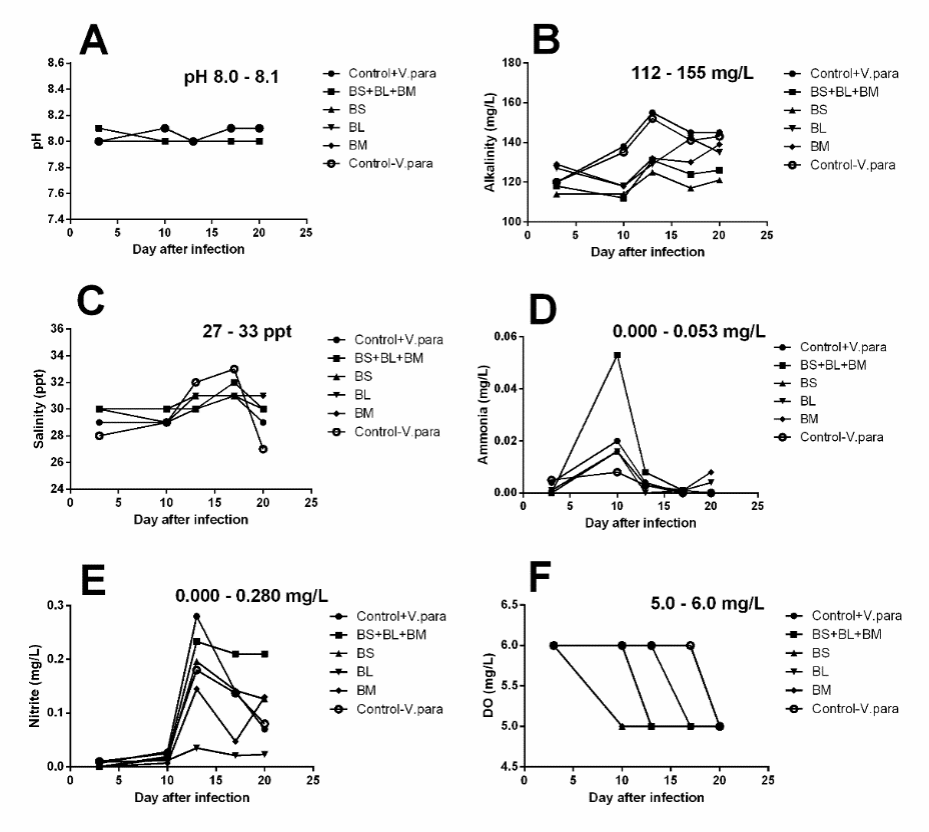
Water quality during the experiment. (A) pH; (B) Alkalinity; (C) Salinity; (D) Ammonia; (E) Nitrite; (F) Dissolved oxygen (DO)

To determine whether the water quality may affect the survival of *Vibrio*-infected shrimp. The pH, alkalinity, salinity, ammonia, nitrite, and dissolved oxygen (DO) were investigated (Fig. 4). The results found that the range of pH, alkalinity, salinity, ammonia, nitrite and DO was 8.0–8.1 (Fig. 4A), 112 mg/L–155 mg/L (Fig. 4B), 27 ppt–33 ppt (Fig. 4C), 0.000 mg/L–0.053 mg/L (Fig. 4D), 0.000 mg/L–0.280 mg/L (Fig. 4E) and 5.0 mg/L–6.0 mg/L (Fig. 4F), respectively. These results were within acceptable values.

## DISCUSSION

The effect of *B. subtilis, B. licheniformis* and *B. megaterium* to control the disease severity of AHPND caused by *Vibrio parahaemolyticus* infection was investigated in white shrimp as a model. White shrimp *(Litopenaeus vannamei)* were infected with *V*. *parahaemolyticus* AHPND strain, subsequently, were fed a diet supplemented with different *Bacillus* strain and their mixed culture. The infected shrimp that was fed with a mixed culture of *Bacillus* strain (*B. subtilis* + *B. licheniformis* + *B. megaterium*), revealed a higher survival rate than those that were fed with a diet containing each of the *Bacillus* strain and non-supplemented control group. This result was supported by the previous study that *B. subtilis, B. licheniformis* and *B. megaterium* were found to inhibit the growth and toxin production of *Vibrio harveyi* resulting in a higher survival rate of shrimp with a diet containing these probiotics than those a control group with non-supplemented (Nakayama *et al*. 2009). The mechanism of this result may be associated with the AHPND pathogenesis of the model in this study.

A previous study by Khimmakthong and Sukkarun (2017) investigated the *V. parahaemolyticus* dissemination in the tissue of *Litopenaeus vannamei*, they found *V. parahaemolyticus* in the gills, hepatopancreas, intestine, muscles and hemolymph. Later, after 6 h of infection, only small amounts of this pathogen were found in the hepatopancreas and intestine with abnormal histopathology. This study suggested that *V. parahaemolyticus* could spread quickly by using the hepatopancreas as the target tissue (Khimmakthong & Sukkarun 2017). Similarly, the present study investigated the dissemination of *V. parahaemolyticus* AHPND strain in the presence of a diet supplemented with different *Bacillus* strain and their mixed culture in a shrimp model. At the endpoint of the experiment (22 days after infection), the infected shrimp without any bacteria contained in a diet (Control+*V. parahaemolyticus*) demonstrated the hepatopancreas, intestine, and muscle could be detected *V. parahaemolyticus* AHPND strain with 100% detection by PCR (Fig. 1C) and revealed the large amount of *Vibrio* spp. viability in all tissues (Fig. 1D). Interestingly, the infected shrimp were fed with a diet supplemented with the mixed culture of *Bacillus* sp. (*B. subtilis* + *B. licheniformis* + *B. megaterium*) showed a lower percentage of *Vibrio* detection by PCR (57.14%) (Fig. 1C) with a small amount of *Vibrio* in hepatopancreas while other groups of infected shrimps reveal higher percent of *Vibrio* detection with a large amount of *Vibrio* viability in all tissues (Fig. 1D). These results suggested that aquaculture of infected shrimp with the mixed culture of *Bacillus* spp. (*B. subtilis* + *B. licheniformis* + *B. megaterium*) in a diet could decrease the severity of AHPND by decreased dissemination of *V. parahaemolyticus* AHPND strain to hepatopancreas which is the target tissue of this disease. Additionally, the infected shrimps with a diet-supplemented *Bacillus* strain and the non-supplement groups were not significantly different in weight. In contrast, the weight of uninfected shrimp was higher than all groups of infected shrimps with significant differences (*p*-value < 0.0001). This result suggested the disease progression in the infected shrimps affected the function of their digestive tract and result in weight loss.

Kyeong-Jun *et al*. (2019) demonstrated the effect of dietary supplementation of three *Bacillus* spp. consist of *B. subtilis, B. pumilus* and *B. licheniformis* on growth performance and disease resistance of *L. vannamei*. They found that shrimp fed with a diet supplemented with only *B. subtilis* had significantly higher growth performance than those feed non-supplement or supplemented with the mixed culture of *B. subtilis* and *B. pumilus* or the mixed culture of three strains (Kyeong-Jun *et al*. 2019). However, different *Bacillus* strains in the mixed culture maybe affect the shrimp’s growth and survival. Recently, Nguyen *et al*. (2021) proposed that the *B. subtilis* DSM33018 strain was shown to degrade AHPND toxins *in vitro*, as detected by Western blots and PirB^VP^ toxin is more susceptible to degradation by this *Bacillus* strain than PirA^VP^ (Nguyen *et al*. 2021). Similarly, the previous study explained the properties of *B. subtilis* which is an exoenzyme-producing bacteria such as protease and amylase that could digest the mucus-coated gram-negative bacterial pathogen. Additionally, *B. subtilis* could produce some antimicrobial molecules that destroy the pathogen’s cell structure, leading to growth inhibition (Vaseeharan & Ramasamy 2003). Moreover, a diet supplemented with *B. subtilis* demonstrated the enhancement of growth and immune response in *L. vannamei* (Shen *et al*. 2010). In contrast, in this present study, the infected shrimp with a diet supplemented only *B. subtilis* (BS) showed lower percent survival with a higher percent *Vibrio*-detected by PCR and higher viability count of *Vibrio* spp. than those supplemented with the mixed culture.

Although a diet supplemented with each strain of *Bacillus* sp. including *B. subtilis, B. licheniformis* revealed no significantly different in survival percentage with the Control+*V. parahaemolyticus* group, except *B*. *megaterium* contained a diet (*p*-value 0.0036). In addition, it seems to slowly decrease the survival percentage when compared to each *Bacillus* strain in this study (Fig. 1A). This result suggested that only one strain of *Bacillus* in this study could not control disease severity and *B*. *megaterium* maybe play a key role to control *V*. *parahaemolyticus* in this infected shrimp model.

*B. megaterium* could be isolated from the digestive tract of *L. vannamei* and was reported the properties of the extracellular enzyme (protease, amylase, lipase) with high antimicrobial production. The *in vivo* study evidenced that a diet supplemented with the *B. megaterium* BM1 strain could be beneficial for the growth of *L. vannamei* by giving a significantly higher specific growth rate compared to other diets (Yuniarti *et al*. 2013). This evidence suggested that *B. megaterium* could be better adaptive in the digestive tract of *L. vannamei* and had beneficial properties as described above.

Additionally, *B. licheniformis* reported the beneficial effects with *L. vannamei* by the number of *Vibrio* spp. was significantly decreased after administration of *B. licheniformis* in *L. vannamei* and revealed improved immune indicated by haemocyte, phenoloxidase and superoxide dismutase were significantly higher than those the control (Li *et al*. 2007). Similarly, Fan *et al*. (2021) reported that pathogen susceptibility and immune suppression in shrimp are caused by nitrite stress which is one of the pollutants commonly found in aquaculture water. They indicated that after nitrite stress of *L. vannamei*, a diet supplemented with *B. licheniformis* revealed improved weight, growth rate, and survival rate (Fan *et al*. 2021)

Although, *B. subtilis, B. licheniformis* and *B. megaterium* reported beneficial properties to *L. vannamei*, however, the result of the present study shows a significant difference in the survival percentage of infected shrimps fed with a diet supplemented with *B. megaterium* only and their mixed culture. However, the mixed culture of these *Bacillus* strains resulted in higher survival percentage of infected *L. vannamei*, the mechanism of the mixed culture of *B. subtilis, B. licheniformis* and *B. megaterium* in controlling *V. parahaemolyticus* AHPND strain in shrimp was required for further exploration of their interaction.

In addition, several studies evident that the genus of *Bacillus* is not only used as an effective probiotic but also used for the treatment of organic waste in aquaculture environments to reduce the risk factor of *V. parahaemolyticus* AHPND strain infection (Kumar *et al*. 2016; Hlordzi *et al*. 2020; Liu *et al*. 2009; Nakayama *et al*. 2009). *B. subtilis, B. licheniformis* and *B. megaterium* have been reported a role in water quality including biochemical oxygen demand (BOD), dissolved oxygen (DO), ammonia, alkalinity and pH (Elsabagh *et al*. 2018; Reddy *et al*. 2018; Cha *et al*. 2013) that demonstrated by the better quality of water in the pond (Kumar *et al*. 2016, Hlordzi *et al*. 2020). The present study used a diet-supplemented *Bacillus* strain to feed infected shrimp, several factors were measured for monitoring water quality during the experiment. The results found that the range of pH, alkalinity, salinity, ammonia, nitrite and DO were within acceptable values.

## CONCLUSION

The present study concluded that a diet supplemented with the mixed culture of *B. subtilis*, *B. licheniformis* and *B. megaterium* could decrease AHPND severity in white shrimp (*L. vannamei*). This mixed culture was supported by higher percent survival, a lower percent of *Vibrio* AHPND strain detected by PCR, and a small amount of *Vibrio* sp. viability count in hepatopancreas than those other groups of infected shrimps. These results suggested that the mixed culture of *Bacillus* spp. in a diet could decrease the severity of AHPND by reducing the dissemination of *V. parahaemolyticus* AHPND strain to hepatopancreas which is the target tissue of this disease. However, further study about the mechanism of the mixed culture from these *Bacillus* strains interacting with *V. parahaemolyticus* AHPND strain in shrimp was required for understanding their interaction.

## Figures and Tables

**Table 1:** Primer used in this study.

Primer name	Sequence (5′-3′)	Product size (bp)
Vp-flaE-79F	5′-GCAGCTGATCAAAACGTTGAGT-3′	897
Vp-flaE-34R	5′-ATTATCGATCGTGCCACTCAC -3′	
TUMSAT-Vp1F	5′-CGCAGATTTGCTTTTGTGAA -3′	500
TUMSAT-Vp1R	5′-AGAAGCTGGCCGAAGTGATA -3′	
TUMSAT-Vp3F	5′-GTGTTGCATAATTTTGTGCA -3′	360
TUMSAT-Vp3R	5′-TTGTACAGAAACCACGACTA -3′	
